# Abdominal paresis and pseudo-hernia secondary to herpes zoster infection: A case report and systematic analysis

**DOI:** 10.1016/j.heliyon.2023.e13578

**Published:** 2023-02-09

**Authors:** Yi Rong Chiew, Chloe Pawa

**Affiliations:** a Department of Neurology, National Neuroscience Institute, Singapore

**Keywords:** Abdominal pseudo-hernia, Herpes zoster infection, Abdominal wall atrophy, Abdominal muscle paresis

## Abstract

**Background:**

Abdominal pseudo-hernia secondary to herpes zoster infection is rare and the clinical features and factors affecting recovery remain poorly understood.

**Aim:**

We aimed to describe the clinical features of patients with abdominal pseudo-hernia secondary to herpes zoster infection and attempt to identify factors associated with poor recovery.

**Design:**

Literature review and retrospective Analysis.

**Methods:**

We report a case and performed a retrospective, systematic review of the demographic background, clinical characteristics and outcomes of patients with abdominal pseudo-hernia secondary to herpes zoster infection in the literature over 20 years (2001–2021).

**Results:**

We analyzed a total of 34 cases. The median age of the patients was 71.5 years. Most of the patients were male (n = 27, 79.4%). The most frequently affected dermatome was T-11 (n = 20, 66.7%). In four (12.5%) patients, abdominal pseudo-hernia started before the onset of rash. In all patients (n = 12, 100%) who underwent nerve conduction study and electromyography, there was electrophysiological evidence of acute denervation. Seven patients (20.6%) had imaging features suggestive of abdominal wall atrophy and denervation. The majority of patients had good recovery. The median follow-up time was 3 (15 days-12 months) months. Patients with pre-existing medical conditions (p = 0.03) were more likely to have a worse recovery.

**Conclusion:**

Abdominal pseudo-hernia is a rare complication of herpes zoster infection with a good prognosis for recovery, although patients with pre-existing disease appear to recover worse. In rare cases, it may occur before the onset of typical zoster rashes and should be suspected, especially in older, male patients with involvement of the lower thoracic dermatomes.

## 1 Introduction

Herpes zoster is an infection caused by reactivation of the varicella zoster virus [1]. After initial infection with the varicella zoster virus, the virus lies dormant in the dorsal root ganglion. In older and immunocompromised patients, immunity to the varicella-zoster virus wanes and there is therefore a risk of viral reactivation causing a herpes zoster infection or commonly known as shingles.

Herpes zoster infection can cause various complications, the most common being postherpetic neuralgia [2]. Cranial nerve palsies, peripheral neuropathies, meningitis, myelitis and vasculopathies can also occur as a result of herpes zoster infection. However, postherpetic abdominal pseudo-hernia is an extremely rare neurological complication of herpes zoster infection that results from paresis of the ipsilateral abdominal muscles following herpes zoster infection. Although the incidence rate of herpes zoster infection is high, only less than 2% of cases of abdominal pseudo-hernia have been reported following herpes zoster infection [3].

## 2 Methods and statistical analysis

We describe the clinical features of our patient who presented with an abdominal pseudo-hernia following herpes zoster infection. Informed consent was obtained from the patient.

We conducted the literature search using PubMed over a 20-year period from 2001 to 2021 using the keywords ‘abdominal pseudo-hernia’, ‘herpes zoster infection’, ‘abdominal wall atrophy’ and ‘abdominal muscle paresis'. The included cases were restricted to articles published in English. Articles written in foreign languages were excluded.

Demographic characteristics, clinical, electrodiagnostic and imaging features, and treatment outcomes of the identified cases were retrospectively analyzed. Categorical variables were summarized as numbers (n) and percentages (%), and statistical analyses were performed using the Pearson chi-square test or Fisher's exact test, with a significant p-value of 0.05.

Continuous variables were expressed as medians (interquartile range) and binomial logistic regression was performed to determine the effect of age on patients' abdominal pseudo-hernia recovery status, with a significant p-value of 0.05.

## 3 Illustrative case

A 65-year-old man with a history of poorly controlled diabetes mellitus presented with a protrusion on the right side of the abdomen that had been present for one month. Seven days before the appearance of the abdominal protrusion, he consulted a general practitioner complaining of right-sided abdominal rash and itching. There were also erythematous rashes and blisters on his right flank. A herpes zoster infection was diagnosed and he was treated with an oral antiviral for seven days.

**Fig. 1:**
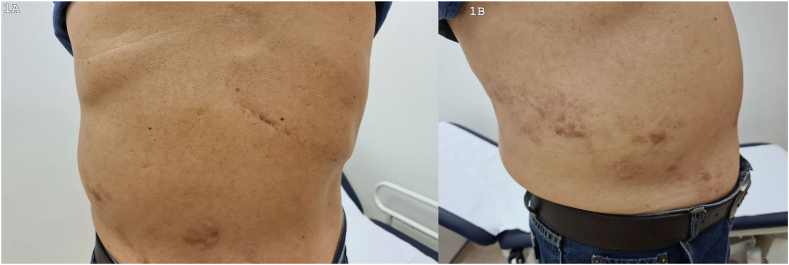
A. Photograph showing right abdominal wall distension. B. Hyperpigmentation of skin overlying the abdominal distension, suggestive of post-herpetic skin rashes.

After completing treatment for the herpes zoster infection, he noticed the abdominal bulge, which was painless. The abdominal bulge was associated with numbness and paraesthesia overlying the abdominal bulge. Clinical examination revealed a non-painful, reducible protrusion measuring 10 × 10 cm in the right flank (Fig. 1(A)). The size of the prominence did not change when the patient stood or coughed. There was reduced pinprick sensation along with allodynia and hyperalgesia and hyperpigmented rashes on the right T9-T11 dermatomes. (Fig. 1(B)). His other neurological and systemic examinations were normal. Ultrasonography of the abdomen was normal. There were no intra-abdominal masses. Electrophysiological examination was not done. A diagnosis of abdominal pseudo-hernia due to herpes zoster infection was made and he was treated conservatively with oral gabapentin for his neuropathic pain. After 3 months of follow-up, his symptoms improved significantly with reduction of the abdominal pseudo-hernia.

## 4 Result of literature review

34 cases of abdominal pseudo-hernia due to herpes zoster infection were included. Their demographic data, clinical features, electrophysiological studies and imaging findings were summarized [[3], [4], [5], [6], [7], [8], [9], [10], [11], [12], [13], [14], [15], [16], [17], [18], [19], [20], [21], [22], [23], [24], [25], [26], [27], [28], [29]]. (Table 1, Table 2) The median age of the patients was 71.5 (43–82) years. Most of the patients were male (n = 27, 79.4%). The median onset of abdominal pseudo-hernia after the eruption of rashes, was 14 (1–60) days. Interestingly, there were four (12.5%) patients in whom abdominal pseudo-hernia occurred before the onset of herpes zoster skin rash. Thoracic dermatome T-11 was most commonly affected (n = 20, 66.7%), followed by T-12 (n = 13, 43.3%), T-10 (n = 12, 40%), T-9 and L-1 (n = 3,10%). There was electrophysiological evidence of acute denervation in all patients (n = 12, 100%) who underwent nerve conduction study and electromyography. Of the patients with available imaging data (n = 30), seven of them (20.6%) had imaging features suggestive of abdominal wall atrophy and denervation. The prognosis of abdominal pseudo-hernia secondary to herpes zoster infection was good. The majority of patients (n = 21, 70%) recovered completely, while 5 patients (16.7%) had significant improvement. The median follow-up time for patients was 3 months.

**Table 1:** Summary of demographics and features of abdominal pseudo-hernia secondary to herpes zoster infection.

Characteristics	Result
Age, median (range), year	71.5 (43–82)
Sex (male: female), n	27: 7
Onset of pseudo-hernia from herpes zoster, median (range), days	14 (1–60)
Onset of pseudo-hernia before herpes zoster rash, n (%)	4 (12.5)
Number of patients who underwent NCS/EMG, n (%)	12 (35.3)
NCS showing denervation (12 patients), n (%)	12 (100)
Imaging Modalities (32 patients)^a^, n (%)	Ultrasound Abdomen: 11 (34.4)
	CT Abdomen: 18 (56.3)
	MRI Abdomen: 2 (6.3)
	MRI lumbosacral spine: 6 (18.8)
Imaging features supportive of abdominal wall atrophy and denervation, n (%)	7 (20.6)
Anatomical involvement (30 patients)^b^, n (%)	T9:3 (10)
	T10: 12 (40)
	T11: 20 (66.7)
	T12: 13 (43.3)
	L1: 3 (10)
Treatment, n (%)	Conservative: 34 (100)
Clinical outcome (30 patients)^c^, n (%)	Full recovery: 21 (70)
	Improve: 5 (16.7)
	No change: 4 (13.3)
Follow-up period, median (range), months	3 (0.5–12)

a Patients 15 and 16 were excluded in view of incomplete documentation.

b Patients 8,9,10 and 12 were excluded in view of incomplete data of imaging.

c Patients 21, 26, 29 and 34 were excluded in view of incomplete documentation.

**Table 2:** Summary of the demographic data, clinical features, investigations and treatment outcomes of patients with abdominal pseudo-hernia secondary to herpes zoster infection.

Patient no.	Ref	Age/Sex	Past Medical History	Herpes Zoster to Pseudo-hernia (days)	Dermatome	Imaging modality	Imaging features of abdominal wall atrophy and denervation	NCS/EMG showing denervation	Treatment	Outcome	Follow up (months)
1	Dobrev et al. (2008)	82/F	No significant history	12	T10-T11	US abdomen	No	Not done	Conservative (Combined vitamin)	Resolution	4
2	Tirelli et al. (2019)	78/F	No significant history	7	T12-L1	CT abdomen	No	Yes	Conservative (Clinical monitoring)	Resolution	12
3	Zuckerman et al. (2001)	67/M	No significant history	2 days BEFORE rash	T9-T10	CT abdomen and US abdomen	No	Yes	Conservative (Clinical monitoring)	Improved	6
4	Yoo et al. (2019)	68/M	HTN, DM	30	T12-L1	CT abdomen	No	Not done	Conservative (Waist band)	Resolution	5
5	Yoo et al. (2019)	75/F	DM	1	T10-T11	CT abdomen	No	Not done	Conservative (Clinical monitoring)	No improvement	2
6	Yoo et al. (2019)	73/M	HTN, DM	20	T11-T12	CT abdomen	No	Not done	Conservative (Clinical monitoring)	Resolution	7
7	Chiu H et al. (2013)	75/M	HTN, DM	60	T10-T11	CT abdomen Thoracolumbar MRI	No	Yes	Conservative (Clinical monitoring)	Improved	7
8	Giuliani et al. (2006)	57/M	Cured colon cancer >5 years prior	5 days BEFORE rash	Not reported	CT abdomen	No	Not done	Conservative (Intravenous betamethasone)	Resolution	1
9	Fujikawa et al. (2019)	66/M	No significant history	1	Not reported	CT abdomen	No	Not done	Conservative (Oral Valaciclovir)	Improved	Unclear
10	Mizumoto et al. (2021)	75/M	No significant history	60	Not reported	US abdomen	No	Not done	Conservative (Oral Valaciclovir)	Resolution	2
11	Azimi SZ et al. (2020)	75/F	No significant history	14	T11-T12	CT abdomen	No	Not done	Conservative (Clinical monitoring)	Resolution	3
12	Shikino et al. (2016)	57/M	DM, HTN	30	Not reported	US and CT abdomen	No	Not done	Conservative (Clinical monitoring)	Improved	1
13	Zaladonis et al. (2021)	76/M	DM, HTN	14	T10-L1	CT abdomen	Yes	Yes	Conservative (Clinical monitoring)	Resolution	8
14	Hung CT et al. (2012)	80/M	No significant history	14	T11-T12	US abdomen	No	Yes	Conservative (Clinical monitoring)	Resolution	2.5
15	Shastry V et al. (2021)	69/M	DM	14	T11-T12	Not reported	Not reported	Not done	Conservative (Clinical monitoring)	No improvement	4
16	Shastry V et al. (2021)	58/F	DM	10 days	T10-11	Not reported	Not reported	Not done	Conservative (Clinical monitoring)	No improvement	4
17	Shastry V et al. (2021)	66/M	HTN	12 days	T11-T12	US abdomen	No	Not done	Conservative (Clinical monitoring)	No improvement	4
18	Eguchi H et al. (2017)	45/M	HTN	unclear	T10-T11	CT abdomen	No	Not done	Conservative (Oral famciclovir)	Resolution	3
19	Mancuso M et al. (2006)	72/M	No significant history	35	T11-T12	US abdomen	No	Yes	Conservative (Oral gabapentin)	Resolution	4.5
20	Yu YH et al. (2019)	58/M	No significant history	7	T9 – T11	US abdomen	No	Not done	Conservative (Oral acyclovir, mecobalamin, Vitamin B1)	Resolution	2
21	Miranda-Merchak et al. (2018)	73/M	No significant history	7	T10-T12	MRI abdomen	Yes	Not done	Conservative (Clinical monitoring)	No documentation	Unclear
22	Mitsutake A et al. (2017)	74/M	No significant history	2	T11	MRI spine	Yes	Yes	Conservative (Clinical monitoring)	Resolution	5
23	Mitsutake A et al. (2017)	48/M	DM	14	T11	MRI spine	No	Yes	Conservative (Clinical monitoring)	Resolution	1.5
24	Mitsutake A et al. (2017)	43/M	No significant history	2 days BEFORE rash	T10	MRI spine	Yes	Yes	Conservative (Clinical monitoring)	Resolution	3
25	Mitsutake A et al. (2017)	74/M	DM	7	T11	MRI spine	Yes	Yes	Conservative (Clinical monitoring)	Resolution	4
26	Tagg NT et al. (2006)	75/M	No significant history	28	T11-T12	MRI abdomen and spine	No	Yes	Conservative (Clinical monitoring)	No documentation	Unclear
27	Oliveira PD et al. (2006)	57/F	No significant history	17	T11-T12	US abdomen	No	Yes	Conservative (Clinical monitoring)	Resolution	3
28	Park SH et al. (2017)	57/M	No significant history	14	T11	CT abdomen	No	Not done	Conservative (Oral antiviral)	Improved	0.5
29	Pulia MS et al. (2011)	77/F	No significant history	14	T12	CT abdomen	Yes	Not done	Nil report	No documentation	Unclear
30	Riopelle A et al. (2019)	72/M	DM/HTN	21	T10-T12	CT abdomen US abdomen	No	Not done	Conservative (Clinical monitoring)	Resolution	Unclear
31	Tan SM et al. (2021)	80/M	DM/HTN	3 days BEFORE rashes	T10-T11	CT abdomen	No	Not done	Conservative (Oral acyclovir)	Resolution	2
32	Tsukita et al. (2019)	71/M	No significant history	No document	T11-T12	CT abdomen	No	Not done	Conservative (Intravenous Acyclovir)	Resolution	Unclear
33	Yagi Y et al. (2017)	68/M	No significant history	6	T11	US abdomen	No	Not done	Conservative (Clinical monitoring)	Resolution	3
34	Yeap E et al. (2021)	71/M	No significant history	60	T9-T10	CT abdomen	Yes	Not done	Conservative (Physiotherapy)	No documentation	Unclear
35	Our patient	65/M	DM	7	T9-T11	US abdomen	No	Not done	Conservative (Clinical monitoring)	Resolution	6

Legend: Ref Reference; F Female; M Male; HTN Hypertension; DM Diabetes Mellitus; US Ultrasound; CT Computed Tomography; MRI Magnetic Resonance Imaging; NCS Nerve Conduction Study; EMG Electromyography.

Subgroup analysis was then performed to identify possible factors that might influence the recovery status of abdominal pseudo-hernia. Patients' age, sex and pre-existing medical conditions were analyzed against their abdominal pseudo-hernia recovery statuses (Table 3, Table 3ba,b). We classified the outcomes of abdominal pseudo-hernia into two groups, improvement to full recovery and no improvement. Patients 21, 26, 29 and 34 were excluded from this analysis due to incomplete documentation of recovery status. Patients with pre-existing medical conditions (p = 0.03) such as diabetes mellitus and hypertension, were more likely to have a worse recovery. There were no statistically significant differences between the two groups in terms of age and sex.

**Table 3:** aAnalysis of factors affecting recovery of abdominal pseudo-hernia.

Factors	Abdominal pseudo-hernia¶		p-value
	Improvement/full recovery (n = 26)	No improvement (n = 4)	
Pre-existing medical condition, n (%)	10 (38.4)	4 (100.0)	0.03
Male gender, n (%)	22 (84.6)	2 (50.0)	NS
Female gender, n (%)	4 (15.4)	2 (50)	NS

**Table 3b:** Analysis of age affecting recovery of abdominal pseudo-hernia using binomial linear regression.

Factors	Abdominal pseudo-hernia		p-value	Odds Ratio	95% Confidence Interval
	Improvement/full recovery (n = 26)	No improvement (n = 4)			
Age, y, median (range)	71.5 (43–82)	67.5 (58–75)	NS	1.0021	(0.9058,1.1088)

¶ Patients 21,26,29 and 34 were excluded due to incomplete documentation of recovery status.

Legend: NS Not significant.

## 5 Discussion

Herpes zoster infections are common, but motor complications are rarely reported after herpes zoster infections. Most literatures reported a prevalence of less than 5%, with Ragozzino et al. reported as low as 1% of patients with motor complications after shingle infections [[30], [31], [32]]. When the virus affects the lower thoracic spine [28] and motor paralysis is confined to the abdominal wall, an abdominal wall bulge mimicking hernia may be the clinical feature.

Differential diagnoses of abdominal bulge include intra-abdominal masses, haematoma and true abdominal hernia [25]. Therefore, radiological imaging of the abdomen is important and should be recommended to rule out these secondary causes requiring surgical intervention. In addition, a study by Miranda-Merchak et al. has shown that the presence of features suggestive of abdominal wall denervation, such as abdominal wall atrophy, signal changes including T2-weighted and STIR (Short Tau Inversion Recovery) hyperintensity of muscles supplied by the particular herpes zoster affected nerve, usually occurring a few weeks after injury, help in the diagnosis of abdominal wall pseudo-hernia [19]. However, in our study, in patients with available imaging data, only seven patients (20.6%) had imaging features of acute denervation. Therefore, it is noteworthy that although a positive MRI finding could potentially help in the diagnosis of abdominal pseudo-hernia, a normal MRI finding does not exclude the diagnosis of this clinical entity.

Abdominal pseudo-hernia due to herpes zoster infection is primarily a clinical diagnosis, based on the timing and correlation of herpes zoster infection with the occurrence of abdominal distension. In our patient, abdominal distension occurred seven days after his shingles infection, which is consistent with most cases reported in the past, in which abdominal distension was preceded by a herpetic rash, with median onset time of one to eight weeks [33]. Rarely, however, abdominal distension can occur before the appearance of the typical herpetic rash, as shown in our study review and the observation by Chernev et al., which complicates the diagnosis [33]. It is therefore very important for clinicians to recognize and identify this clinical entity. The absence of rashes should not lead to false reassurance and one should not rule out the diagnosis of postherpetic abdominal pseudo-hernia in the absence of herpetic rashes.

Our patient presented with T9 to T11 dermatome abdominal pseudo-hernia, similar to our review which showed that this clinical entity most frequently affected the T11 dermatome. We also found that the majority of patients were elderly male, with a median age of 71.5 years. These results are also consistent with the observations of Chernev et al. [33].

According to our literature review, electrophysiological studies were not routinely performed in patients diagnosed with abdominal pseudo-hernia secondary to herpes zoster infection. However, our study found that all patients in whom nerve conduction studies and electromyography were performed had features of acute denervation. This may suggest that nerve conduction studies and electromyography of the thoraco-lumbar paraspinal muscles, are useful in diagnosing abdominal wall paresis leading to abdominal wall pseudo-hernia following herpes infection. Unfortunately, the utility of electrophysiological studies in the diagnosis of this clinical entity may not be accurately reflected given the small sample size. Therefore, larger and prospective studies will be useful in the future to clarify this issue.

The treatment of abdominal pseudo-hernia is primarily conservative and the prognosis is reported to be good in the literature [[3], [4], [5],[8], [9], [10], [11], [12], [13], [14],[16], [17], [18],^20^,[22], [23], [24], [25], [26], [27], [28]]. Our study showed that the majority of patients (n = 21, 70%) recovered completely. However, as far as we know, our study is the first study that has conducted a statistical analysis with the aim of identifying the factors that may lead to poor recovery in a proportion of patients. We found that patients with pre-existing conditions such as diabetes mellitus and hypertension were more likely to have a poorer recovery. However, our study is limited by its retrospective nature and small sample size. In addition, control of patients' underlying medical conditions, such as HbA1c level in diabetes mellitus, has not been clearly documented in the various literatures, making it difficult to establish an association between pre-existing medical conditions and the healing outcome of abdominal pseudo-hernia. This is a topic that would benefit from further clarification by reporting and analyzing more clinical data in the future.

In conclusion, abdominal pseudo-hernia is a rare neurological complication of herpes zoster infection resulting from isolated abdominal wall paresis. It is important to recognize this clinical entity as the prognosis is good with conservative management and no surgical intervention is required, although patients with pre-existing conditions may have a poorer recovery. Abdominal pseudo-hernia may rarely occur before the onset of typical zoster rashes. Therefore, continuous surveillance for zoster infection is recommended after the occurrence of abdominal pseudo-hernia, and it should be suspected especially in elderly male patients with involvement of the lower thoracic dermatomes.

## Declarations

### Author contribution statement

Yi Rong Chiew, Chloe Pawa: Conceived and designed the experiments; Analyzed and interpreted the data; Contributed reagents, materials, analysis tools or data; Wrote the paper.

### Funding statement

This research did not receive any specific grant from funding agencies in the public, commercial, or not-for-profit sectors.

### Data availability statement

Data will be made available on request.

## Availability of data and materials

We documented the patient's data reported in the article. We will share the de-identified data on reasonable request. To request the data, please contact the corresponding author.

## Declaration of competing interest

The authors declare that they have no known competing financial interests or personal relationships that could have appeared to influence the work reported in this paper.
